# Brain Vitamin E Deficiency During Development Is Associated With Increased Glutamate Levels and Anxiety in Adult Mice

**DOI:** 10.3389/fnbeh.2018.00310

**Published:** 2018-12-11

**Authors:** Catherine M. Desrumaux, Marine Mansuy, Stéphanie Lemaire, Justine Przybilski, Naig Le Guern, Laurent Givalois, Laurent Lagrost

**Affiliations:** ^1^INSERM, U1198, Team “Environmental Impacts in Alzheimer’s Disease and Related Disorders” (EiAlz), Montpellier, France; ^2^Faculty of Sciences, Université Montpellier, Montpellier, France; ^3^EPHE, Paris, France; ^4^LipSTIC LabEx, Fondation de Coopération Scientifique Bourgogne-Franche Comté, Dijon, France; ^5^INSERM, LNC UMR1231, Dijon, France; ^6^University Bourgogne Franche-Comté, LNC UMR1231, Dijon, France; ^7^Hôpital du Bocage, Dijon, France

**Keywords:** vitamin E, lipid transfer, development, brain, glutamate

## Abstract

Vitamin E, the most important lipophilic radical scavenging antioxidant *in vivo*, has a pivotal role in brain. In an earlier study, we observed that adult mice with a defect in the gene encoding plasma phospholipid transfer protein (PLTP) display a moderate reduction in cerebral vitamin E levels, and exacerbated anxiety despite normal locomotion and memory functions. Here we sought to determine whether dietary vitamin E supplementation can modulate neurotransmitter levels and alleviate the increased anxiety phenotype of PLTP-deficient (*PLTP*^−/−^) mice. To address this question, a vitamin E-enriched diet was used, and two complementary approches were implemented: (i) “early supplementation”: neurotransmitter levels and anxiety were assessed in 6 months old *PLTP*^−/−^ mice born from vitamin E-supplemented parents; and (ii) “late supplementation”: neurotransmitter levels and anxiety were assessed in 6 months old *PLTP*^−/−^ mice fed a vitamin E-enriched diet from weaning. Our results show for the first time that an inadequate supply of vitamin E during development, due to moderate maternal vitamin E deficiency, is associated with reduced brain vitamin E levels at birth and irreversible alterations in brain glutamate levels. They also suggest this deficiency is associated with increased anxiety at adulthood. Thus, the present study leads to conclude on the importance of the micronutrient vitamin E during pregnancy.

## Introduction

Vitamin E acts as the most important lipophilic radical scavenging antioxidant *in vivo* (Niki, 2014). Among the eight forms of vitamin E, alpha-tocopherol is transported into the circulation by the alpha-tocopherol transfer protein (alpha-TTP) with much higher efficiency than the others, and is the most active form of vitamin E in mammals. In addition to its potent antioxidant action, alpha-tocopherol can act as a gene regulation molecule, and its incorporation into cell membranes can influence the activity of membrane-associated and integrated proteins thus modulating signaling pathways (Galli et al., 2017).

**Figure 1:**
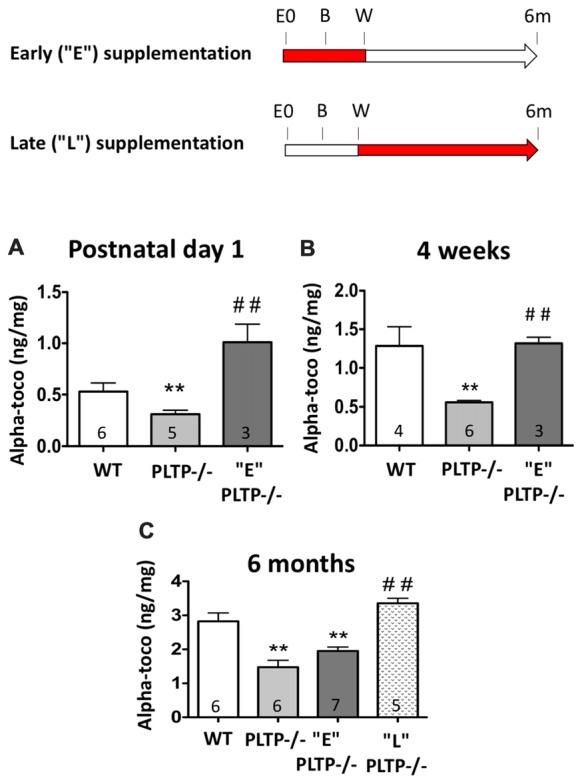
Procedures followed for vitamin E supplementation of plasma phospholipid transfer protein (PLTP)-deficient mice, and cerebral alpha-tocopherol levels in wild-type (WT) mice and PLTP-deficient mice supplemented or not with vitamin E. Top panel: early supplementation (“E”): *PLTP*^−/−^ breeding mice were fed a vitamin E-enriched diet and anxiety levels were measured in their progeny (“E” *PLTP*^−/−^ mice) at the age of 6 months. Late supplementation (“L”): *PLTP*^−/−^ mice were fed a vitamin E-enriched diet from weaning and their anxiety level was determined at the age of 6 months. E0, embryonic day 0; B, birth; W, weaning; 6 m, 6 months. The red portions of the arrows in the experimental timeline indicate the timing of vitamin E supplementation. Lower panel: alpha-tocopherol levels were measured in brain homogenates from WT mice, *PLTP*^−/−^ mice and *PLTP*^−/−^ mice born from vitamin E-supplemented dams at post-natal day 1 **(A)** or at the time of weaning **(B)**. **(C)** Alpha-tocopherol levels were measured in brain homogenates from 6-month old WT mice, *PLTP*^−/−^ mice, *PLTP*^−/−^ mice fed a vitamin E-supplemented diet from weaning, and *PLTP*^−/−^ mice born from vitamin E-supplemented dams. ANOVA for “postnatal day 1” data: *F*_(2,11)_ = 11.77, *p* < 0.01; ANOVA for “4 weeks” data: *F*_(2,11)_ = 11.33, *p* < 0.01; ANOVA for 6 months’ data: *F*_(3,20)_ = 21.15, *p* < 0.001. **p < 0.01 vs. *WT*, ^##^*p* < 0.01 vs. *PLTP*^−/−^ mice (Newman Keuls’ multiple comparison). The number of mice in each group is indicated on the graph.

Vitamin E has a pivotal role in brain. The pathological manifestations of the familial syndrome ataxia with vitamin E deficiency (AVED), caused by mutations in the alpha-TTP gene in humans, and also observed in alpha-TTP-knockout mice, are represented by severe neurological symptoms (Di Donato et al., 2010) that can be reversed by α-tocopherol supplementation (Yokota et al., 2001). Alpha-TTP-deficient mice, in which the reduction of vitamin E content in tissues is drastic and ubiquitous, were also shown to display an increased anxiety phenotype (Yokota et al., 2001). Dietary vitamin E deficiency was reported to increase anxiety-like behavior in juvenile and adult rats as well (Terada et al., 2011). In an earlier study, we observed that adult mice with a defect in the gene encoding another determinant of vitamin E transport, i.e., plasma phospholipid transfer protein (PLTP), display a moderate reduction in cerebral vitamin E levels, and exacerbated anxiety despite normal locomotion and memory functions (Desrumaux et al., 2005). Together, these reports suggested a role of vitamin E in the etiology of anxiety. In the present study, we sought to determine whether dietary vitamin E supplementation can modulate neurotransmitter levels and alleviate the increased anxiety phenotype of PLTP-deficient (*PLTP^−/–^)* mice. To address this question, a vitamin E-enriched diet (alpha-tocopherol 800 mg/kg diet) was used, and two complementary approches were implemented: (i) “early supplementation”: neurotransmitter levels and anxiety were assessed in 6 months old *PLTP*^−/−^ mice born from vitamin E-supplemented parents (“E” group); and (ii) “late supplementation”: neurotransmitter levels and anxiety were assessed in 6 months old *PLTP*^−/−^ mice fed a vitamin E-enriched diet from weaning (“L” group; Figure 1).

## Materials and Methods

### Mice

PLTP knocked-out (*PLTP*^−/−^) and wild-type (*WT*) C57BL/6 mouse females were used in the present study. Newborn mice from both sexes were used for alpha-tocopherol assays. *PLTP*^−/−^ mice were a kind gift from Dr. X. C. Jiang’s laboratory (SUNY Downstate Medical Center, New York, NY, USA). Mice were housed five per cage and fed a standard chow diet (A03 diet, Safe, Augy, France) or a vitamin E-supplemented chow diet (A03 containing 800 mg/kg α-tocopherol acetate, Safe, Augy, France). Behavioral experiments were performed between 10:00 AM and 05:00 PM. All animal procedures were conducted in strict adherence of the EU Directive 86-609, modified by the decrees 87-848 and 2001-464, and ethics approval was acquired from the “Comité d’Ethique de l’Université de Bourgogne,” under the reference 1305.

### Alpha-Tocopherol Assay

Mice were sacrificed by decapitation, and their whole brain was homogenized. Alpha-tocopherol was assayed by liquid chromatography-mass spectrometry (LC-MS), as previously described (Desrumaux et al., 2005), in milk spots from newborn mice and in brain homogenates.

### Neurotransmitter Assays

Glutamate and gamma aminobutyric acid (GABA) concentrations were measured by high-pressure liquid chromatography (HPLC). In brief, 200 μL of brain homogenates was subjected to a deproteinization step using 30% sulfosalicylic acid solution (Sigma). Then, supernatants were diluted with Jeol sampling buffer (JEOL) containing 0.2 μmol/mL of aminoethylcysteine and glucosaminic acid (internal standards; Sigma). Supernatants (50 μL) were then injected into an automated amino acid analyzer (JEOL Aminotac 500; Tokyo, Japan) and eluted with lithium citrate buffers. Glutamate and GABA were detected at 570 nm. Data acquisition and calculations were performed using the JEOL Workstations software.

Serotonin concentrations were measured by HPLC with fluorescence detection (Waters). In brief, 500 μL of brain homogenates was subjected to a deproteinization step using perchloric acid 4 M (Riedel-de Haen). The chromatography was achieved using C18 LiChroCART 250-4 LicChrospher 100 RP-18 (5 μm) column (Merck). The mobile phase, delivered at 1 ml/min flow rate was as follow: 50 mM sodium acetate, 20 mM citric acid, 4 mM octane sulfonic acid, 0.17 mM EDTA and 1 mM dibutylamine (Sigma) plus 20% methanol and filtered through a 0.22 μm Millipore filter. The fluorescence detector settings were the following: excitation = 302 nm; emission = 340 nm. The sample injection volume was 20 μL. Standards solutions at known concentrations were daily injected into the system and 5-fluoro-DL-tryptophane used as internal standard.

### Assessment of Anxiety

Anxiety levels were assessed in a blind fashion with an Elevated Plus-Maze, as previously described (Desrumaux et al., 2005).

### Statistical Analysis

Results are expressed as mean ± SEM. The statistical significance of differences between data means was determined using a Student’s *t*-test or a one-way ANOVA followed by Newman Keul’s *post hoc* analysis, as appropriate.

## Results

### Alpha-Tocopherol Levels in WT and PLTP^−/−^ Brains and Impact of Vitamin E Supplementation

Alpha-tocopherol levels were measured in brain homogenates from 1-day old *WT*, *PLTP*^−/−^ mice and “E” *PLTP*^−/−^ mice. As shown in Figure 1A, PLTP deficiency is associated with a significant decrease in the alpha-tocopherol content of the brain at birth (−42%, *p* < 0.01). In “E” PLTP-deficient animals, a marked, 3.3-fold increase in cerebral alpha-tocopherol content was measured (*p* < 0.01 vs. *PLTP*^−/−^ mice), and levels were about 2-fold higher than those measured in brain samples from *WT* mice. Interestingly, when alpha-tocopherol was assayed in milk spot extracts, the opposite was observed: milk obtained from *PLTP*^−/−^ mice was enriched in alpha-tocopherol compared to that obtained from *WT* mice, and a decrease in milk alpha-tocopherol content was measured in “E” *PLTP*^−/−^ mice (Supplementary Figure S1). In brain homogenates from 4-week-old mice (Figure 1B), a marked reduction of the alpha-tocopherol level was again measured in *PLTP*^−/−^ mice compared to *WT* mice (−57%, *p* < 0.01). In “E” *PLTP*^−/−^ mice, brain alpha-tocopherol levels were 2.4-fold higher than those in *PLTP*^−/−^ mice, and were comparable to those measured in *WT* brains. At the age of 6 months (Figure 1C), a 47.5% reduction of the brain alpha-tocopherol content was measured in *PLTP*^−/−^ mice compared to *WT* mice (*p* < 0.01). In “L” *PLTP*^−/−^ mice, brain alpha-tocopherol levels were 2.3-fold higher than in *PLTP*^−/−^ mice and were not significantly different from those measured in *WT* mice. In “E” *PLTP*^−/−^ mice, brain alpha-tocopherol levels at 6 months were 30.9% lower than those of *WT* mice, and were not significantly different from those of *PLTP*^−/−^ mice. Thus, it appears that between birth and the age of 6 months, brain alpha-tocopherol content in the “E” group decreases relative to *WT*, indicating that the effect of the *in utero* supplementation fades progressively after birth and is not visible anymore at the age of 6 months. In contrast, in the “L” group the effect of vitamin E supplementation is highly visible at 6 months.

### Neurotransmitter Levels in WT and PLTP^−/−^ Brains and Impact of Early “E” or Late “L” Vitamin E Supplementation

**Figure 2:**
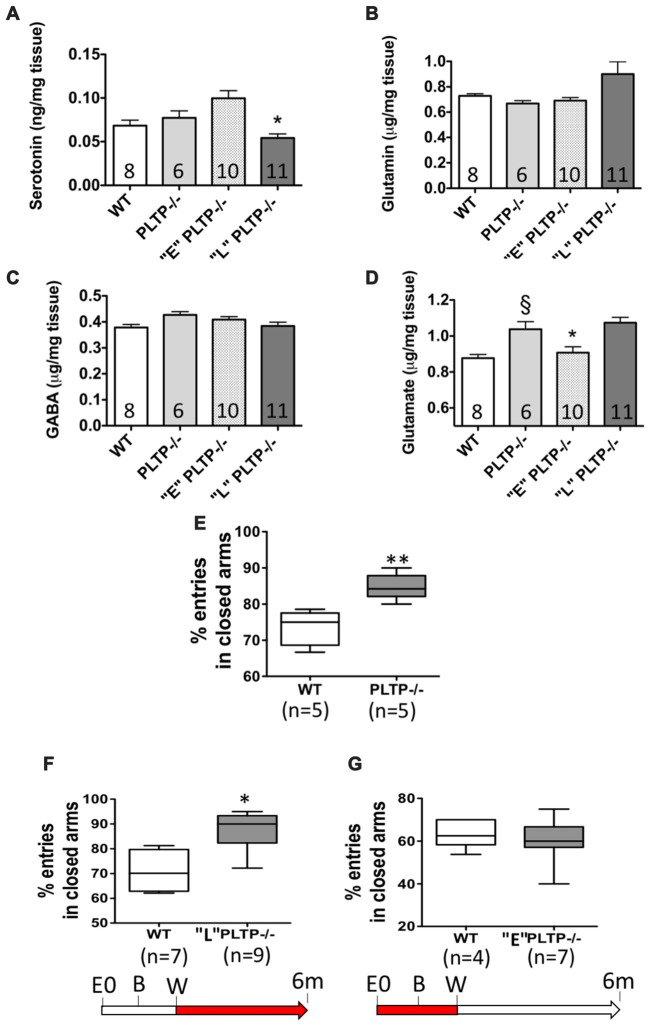
Neurotransmitter and anxiety levels in 6-month-old WT mice and PLTP-deficient mice supplemented or not with vitamin E. Serotonin **(A)**, glutamin **(B)**, gamma aminobutyric acid (GABA; **C**) and glutamate **(D)** levels were assayed by high-pressure liquid chromatography (HPLC) in brain homogenates from *WT*, *PLTP*^−/−^, “E” *PLTP*^−/−^ and “L” *PLTP*^−/−^ mice. ANOVA for serotonin levels: *F*_(3, 31)_ = 8.57, *p* < 0.001; ANOVA for glutamate levels: *F*_(3,31)_ = 9.76, *p* < 0.0001. *p < 0.05 vs. *PLTP*^−/−^, ^§^*p* < 0.01 vs. *WT* mice (Newman Keuls’ multiple comparison test). Anxiety levels were measured in the Elevated Plus-Maze in *WT* mice and *PLTP*^−/−^ mice **(E)**, “L” *PLTP*^−/−^ mice **(F)**, or “E” *PLTP*^−/−^ mice **(G)**. The red portions of the arrows in the experimental timeline indicate the timing of vitamin E supplementation. The number of animals in each group is indicated on the graph. *p < 0.05, **p < 0.01 vs. *WT* mice (Student’s *t*-test).

Serotonin, glutamin, glutamate and GABA levels were measured in brain homogenates from 6 month-old *WT* mice, *PLTP*^−/−^ mice, “E” *PLTP*^−/−^ mice and “L” *PLTP*^−/−^ mice. As shown in Figure 2, no significant difference in serotonin, glutamin and GABA levels was observed between brain samples of *PLTP*^−/−^ and *WT* mice. Dietary supplementation with vitamin E from weaning (“L” group), but not maternal vitamin E supplementation (“E” group), led to a reduction of the serotonin level in brain homogenates from *PLTP*^−/−^ mice (Figure 2A). As shown in Figure 2D, a significant increase in glutamate level was measured in brain samples of *PLTP*^−/−^ mice compared to *WT* mice. Interestingly, maternal vitamin E supplementation led to a significant reduction of the glutamate level in brain homogenates from *PLTP*^−/−^ mice (“E” group), while dietary supplementation with vitamin E from weaning (“L” group) had no effect.

### Anxiety Levels in WT and PLTP^−/−^ Brains and Impact of Early “E” or Late “L” Vitamin E Supplementation

When subjected to the elevated plus-maze paradigm, *PLTP*^−/−^ made more entries in the closed arms compared to *WT* mice (84.8 ± 1.6% vs. 73.5 ± 2.1%, *p* < 0.01; Figure 2E). However, unlike alpha-TTP-deficient mice in which the systemic and complete alpha-tocopherol deficient trait led to abnormal motor performance and severe ataxia in addition to anxiety (Yokota et al., 2001), *PLTP*^−/−^ mice showed no evidence of disorders in activity and neuromotor coordination (Desrumaux et al., 2005). We next measured anxiety levels in 6 months old *PLTP*^−/−^ mice born from vitamin E-supplemented parents and fed a vitamin E-enriched diet from weaning. Interestingly, anxiety levels in these mice were comparable to those measured in *WT* mice (73.1 ± 5.6% entries in closed arms compared to 71.5 ± 5.6% in *WT* mice; data not shown). To distinguish the impact of early vitamin E supplementation from that of late supplementation, we compared anxiety levels in “E” *PLTP*^−/−^ mice, in “L” *PLTP*^−/−^ mice, and in *WT* mice. As shown in Figures 2F,G, anxiety levels in “L” mice were still higher than those of *WT* mice (87.5 ± 3.0% entries in closed arms compared to 70.9 ± 4.4% in *WT* mice, *p* < 0.05). In contrast, “E” *PLTP*^−/−^ mice displayed a normal anxiety level (60.3 ± 3.2% entries in closed arms compared to 63.1 ± 2.2% in *WT* mice).

## Discussion

Previous studies demonstrated that a systemic vitamin E deficiency in mice and humans, resulting from mutation in the alpha-TTP gene, is associated with neurological dysfunctions and increased anxiety (Ouahchi et al., 1995; Yokota et al., 2001; Gohil et al., 2003). In the present study, and as reported earlier by our group (Desrumaux et al., 2005), *PLTP*^−/−^ mice were characterized by a 30% reduced cerebral vitamin E level and increased anxiety, as demonstrated using the Elevated Plus-Maze test. Since the cognitive and neurophysiological dysfunction that characterizes anxiety disorders has been linked to regional dysregulation of excitatory/inhibitory neurobiological pathways (Etkin and Wager, 2007; Martin et al., 2010), we investigated the possible involvement of different neurotransmitter systems in the anxiogenic action of vitamin E deficit in our *PLTP*^−/−^ mice. A significant increase in brain glutamate levels was measured in *PLTP*^−/−^ mice compared to *WT* mice, and was completely restored through maternal (“E” group), but not dietary (“L” group) vitamin E supplementation. Earlier studies reported the implication of vitamin E in brain structures and neurotransmitter systems that are important for anxiety. For example, vitamin E can enhance binding of the GABA_A_ receptor (Takahashi et al., 1984), which could conceivably ameliorate anxiety since reduction of such binding has been reported to produce benzodiazepine-resistant anxiety in mice (Sibille et al., 2000). Vitamin E may also normalize brain serotonin (Lee et al., 2001) that has been implicated in some forms of human anxiety because of decreases in the state when treated with serotonin re-uptake inhibitors (Naughton et al., 2000). Results from the present study suggest that the impact of vitamin E deficit on anxiety levels in our *PLTP*^−/−^ mice might relate on alterations in brain glutamate levels. As the most important excitatory neurotransmitter in the brain, glutamate plays a major role in brain function, from the first stages of neurogenesis until cerebral aging, and a dysfunction of glutamatergic transmission is involved in many neurological diseases as well as mental illnesses. Several lines of evidence from animal studies have implicated the glutamatergic system, including ionotropic and metabotropic glutamate (mGlu) receptors, in emotional-affective behaviors as well as in the pathophysiology of anxiety disorders (Ferraguti, 2018). Recently, Hartmann et al. (2017) reported that forebrain glutamatergic neurons mediate the anxiogenic effects of the glucocorticoid receptor. Based on our observations, the relationship between brain glutamate levels during development and anxiety in later life would deserve further investigation.

Although they warrant to be strengthened using additional paradigms, our results obtained using the Elevated-Plus Maze suggest for the first time that prenatal and/or early post-natal vitamin E supplementation allows to restore normal anxiety levels in *PLTP*^−/−^ mice, while dietary supplementation after weaning does not. It is worthy of note that anxiety was restored as well in a group of mice supplemented *in utero*, during suckling and after weaning. Together, these findings suggest that during development and/or early life, vitamin E plays a role in the establishment of neuronal circuits that determine anxiety. Although potentially deleterious effects of supra-nutritional doses of vitamin E during pregnancy on spatial learning in the adult offspring have been pointed out (Ambrogini et al., 2011; Betti et al., 2011), it is worthy of note that in these studies, the dose used for dietary supplementation was 20 times higher than in the present study.

Vitamin E is the most abundant lipophilic antioxidant in the brain. Recently, a possible association between oxidative stress and several mental disorders including schizophrenia, depression, anxiety and bipolar disorder has been suggested (Andreazza, 2012). For instance, Berry et al. (2007) demonstrated that generalized low oxidative stress is associated with reduced anxiety in mice with a targeted mutation of the gene encoding the life span determinant p66(Shc). Concordantly, a correlation between the activity of antioxidative enzymes (glyoxalase 1, glutathione reductase 1) and an anxiety phenotype was found in a study using six inbred mouse strains (Hovatta et al., 2005). Acute and chronic vitamin A supplementation induces anxiety-like behavior and oxidative/nitrosative stress in the adult rat hippocampus, substantia nigra and striatum (Schnorr et al., 2015). Ovariectomy causes oxidative stress in different central nervous system structures owing to depletion of antioxidant content leading to an anxiogenic profile (Da Silva Morrone et al., 2016). In human populations, anxiolytic effect of dietary antioxidants (Boldrini et al., 2018) and a positive correlation between peripheral blood oxidative stress markers and anxiety behavior (Steenkamp et al., 2017) have been reported as well. Thus, the anxiolytic effect of vitamin E in rodents and human populations may be ascribed to its antioxidant properties. It is not unlikely however that the vitamin E’s anxiolytic effects might arise from some influence other than its antioxidant properties alone, such as its ability to modulate protein kinase C (PKC) activity. Betti et al. (2011) reported that rats born from dams supplemented with high doses of alpha-tocopherol through the diet (1 g/kg/day) over pregnancy and lactation showed PKC activity inhibition up to weaning. In adulthood, i.e., when behavioral tests were conducted, rats exhibited a fully recovered PKC activity, but reduced synpatic plasticity and long-term spatial memory; therefore, the observed behavioral changes are likely to relate to permanent alterations that occurred during brain development under PKC activity inhibition (Betti et al., 2011). Whether the impact of vitamin E on anxiety is related to its antioxidant or non-antioxidant properties remains to be determined.

Evidence from both epidemiological studies and animal models indicates that maternal diet and metabolic status play a critical role in programming the neural circuitry that regulates emotional behavior, resulting in long-term consequences for the offspring. In particular, the impact of high-fat diet feeding on the anxiety state in the offspring has been studied quite extensively. Our results suggest for the first time that an inadequate supply of vitamin E during development and/or early life, due to moderate maternal vitamin E deficiency, is associated with reduced brain vitamin E levels at birth, and increased anxiety at adulthood. Interestingly, our study also highlights the fact that PLTP plays a major role in vitamin E distribution between biological fluids and tissues: while a 30% decrease in brain vitamin E level is measured in *PLTP*^−/−^ mice, and is restored by dietary vitamin E supplementation, opposite variations are observed in the milk of lactating females.

Thus, the present study suggests the importance of the micronutrient vitamin E during pregnancy to prevent increased anxiety in later life.

## Author Contributions

MM, SL, JP and CD performed the experiments. NLG performed mice breeding. LG and SL corrected the manuscript. LL and CD designed the study, analyzed the data and wrote the manuscript.

## Conflict of Interest Statement

The authors declare that the research was conducted in the absence of any commercial or financial relationships that could be construed as a potential conflict of interest.

## Supplementary Material

FIGURE S1 Alpha-tocopherol levels in milk spot extracts. Alpha-tocopherol levels were measured in milk spot extracts from WT mice, *PLTP*^−/−^ mice and *PLTP*^−/−^ mice born from vitamin E-supplemented dams at postnatal day 1. The number of mice in each group is indicated on the graph.

## Abbreviations

Alpha-TTP: alpha-tocopherol transfer protein
AVED: ataxia with vitamin E deficiency
B: birth
E0: embryonic day 0
GABA: gamma aminobutyric acid
mGlu: metabotropic glutamate
PKC: protein kinase C
PLTP: phospholipid transfer protein
W: weaning
WT: wild-type.
