# Supplementary figures and images for: Brain Vitamin E Deficiency During Development Is Associated With Increased Glutamate Levels and Anxiety in Adult Mice
Source: Front Behav Neurosci. 2018 Dec 11;12:310. doi: 10.3389/fnbeh.2018.00310 (PMC6297247; doi:10.3389/fnbeh.2018.00310)

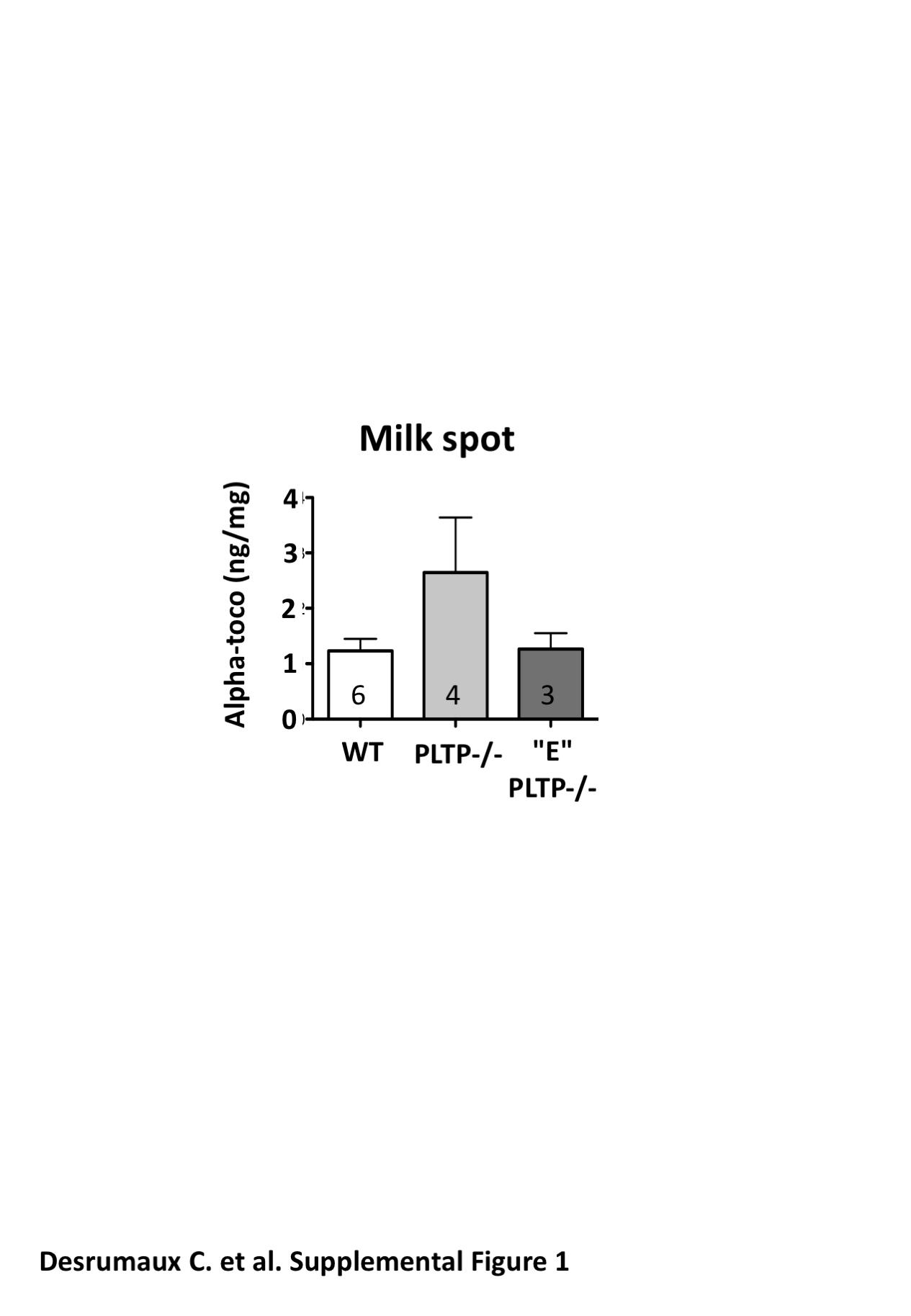

Supplement: FIGURE S1 — Alpha-tocopherol levels in milk spot extracts. Alpha-tocopherol levels were measured in milk spot extracts from WT mice, PLTP−/− mice and PLTP−/− mice born from vitamin E-supplemented dams at postnatal day 1. The number of mice in each group is indicated on the graph. [file Image_1.TIFF]
